# Increased expression of the long non-coding RNA ANRIL promotes lung cancer cell metastasis and correlates with poor prognosis

**DOI:** 10.1186/s13000-015-0247-7

**Published:** 2015-03-27

**Authors:** Ling Lin, Zhi-Tao Gu, Wen-Hu Chen, Ke-Jian Cao

**Affiliations:** Department of Thoracic Surgery, Shanghai Chest Hospital, Shanghai Jiaotong University, 241 West Huaihai Road, Shanghai, 200030 China

**Keywords:** Non-small cell lung cancer, Long non-coding RNAs, ANRIL, Prognosis

## Abstract

**Background:**

Emerging evidences indicate that dysregulated long non-coding RNAs (lncRNAs) are implicated in cancer tumorigenesis and progression. LncRNA ANRIL has been shown to promote the progression of gastric cancer. However, the role of lncRNA ANRIL in human non-small cell lung cancer (NSCLC) remains unclear.

**Methods:**

Expression of lncRNA ANRIL was analyzed in 87 NSCLC tissues and three lung cancer cell lines by quantitative real-time PCR (qRT-PCR). The correlation of lncRNA ANRIL with clinicopathological features and prognosis was analyzed. Suppression of lncRNA ANRIL using siRNA treatment was performed in order to explore its role in tumor progression.

**Results:**

The expression level of lncRNA ANRIL was higher in NSCLC tissues and lung cancer cells than in adjacent non-tumor tissues and normal human bronchial epithelial cells. Higher expression of lncRNA ANRIL in NSCLC tissues was associated with higher TNM stage and advanced lymph node metastasis. Patients with high lncRNA ANRIL expression had poorer overall survival compared with low lncRNA ANRIL group. Univariate and multivariate analyses suggested that high expression of lncRNA ANRIL was an independent poor prognostic indicator for NSCLC patients. Moreover, knockdown of lncRNA ANRIL expression could inhibit lung cancer cell proliferation, migration and invasion in vitro.

**Conclusions:**

Our results suggested that lncRNA ANRIL was a potential biomarker for NSCLC prognosis, and the dysregulation of lncRNA ANRIL may play an important role in NSCLC progression.

**Virtual Slides:**

The virtual slide(s) for this article can be found here: http://www.diagnosticpathology.diagnomx.eu/vs/1707061287149690.

## Background

Lung cancer is one of the leading causes of all cancer-related deaths worldwide, and with an incidence of over 200000 new cases every year [1]. Approximately, 85% of all lung cancer cases are categorized as non-small cell lung cancer (NSCLC), and more than 50% of NSCLC patients have advanced local invasion and/or distant metastases [2]. Despite much progress in early detection and treatment, the 5-year survival rate for NSCLC patients at later stages is only 5-20% [3]. Therefore, it is necessary for us to discover the underlying molecular mechanisms and screen useful biomarkers and novel therapeutic targets of NSCLC.

Traditionally, cancer was regarded as a genetic disease, but current research revealed that cancer development and progression involves epigenetic abnormalities [4]. Genetic continuity has been shown to involve epigenetic regulation such as DNA methylation, histone deacetylation and non-coding RNA (ncRNA) regulation [5]. Long non-coding RNAs (lncRNAs) are more than 200 nucleotides in length with limited or no protein-coding capacity and serve as the primary regulatory ncRNA [6]. Increasing evidences showed that lncRNAs could play an important role in cellular development, differentiation, and many other biological processes [7-9].

Polycomb repressive complex 2 (PRC2) contained the core subunits SUZ12, EED and EZH2 and regulated transcription by establishing di- and trimethylation of histone H3 lysine 27 (H3K27me2 and H3K27me3), critical epigenetic silencing marks [10]. PRC2 target genes played key roles in cell cycle regulation, stem cell self-renewal and cell fate decisions and are frequently targeted for epigenetic modulation in cancer [11]. Recent studies showed that PRC2 could act as an oncogene or tumor suppressor in tumors [12]. lncRNA ANRIL was reported to have a direct role in recruiting PRC2 complexes to specific loci and repress gene expression [13].

ANRIL (antisense non-coding RNA in the INK4 locus), a 3.8 kb lncRNA expressed in the opposite direction from INK4A-ARF-INK4B gene cluster [14]. Common disease genomewide association studies showed ANRIL gene as a genetic susceptibility locus shared associated by coronary disease, intracranial aneurysm, type 2 diabetes and also cancers [15]. Yap et al. found that lncRNA ANRIL was significantly up-regulated in prostate cancer and involved in repressing of the p15/CDKN2B-p16/CDKN2Ap14/ARF gene cluster in Cis by directly binding to the PRC [16]. Zhang et al. showed that lncRNA ANRIL was increased in gastric cancer and associated with tumor size and advanced TNM stage. Further experiments revealed that ANRIL knockdown significantly repressed the proliferation both in vitro and in vivo [17]. However, lncRNA ANRIL expression in NSCLC and the underlying mechanism is still unknown.

In this study, the biological functions of lncRNA ANRIL in NSCLC development were explored by examining the expression pattern of lncRNA ANRIL in NSCLC tissues. Moreover, analysis of existent association with both clinicopathological features and prognosis were examined in order to determine whether lncRNA ANRIL could be considered a potential prognostic factor for the prediction of clinical outcomes in NSCLC patients. Finally, we conducted in vitro assay to demonstrate the biological functions of lncRNA ANRIL on NSCLC progression.

## Methods

### Patients and specimens

A total of 87 primary NSCLC patients were collected from the Department of General Thoracic Surgery, Shanghai Chest Hospital between 2004 and 2006. All patients did not receive chemotherapy or radiotherapy prior to surgery. The clinicopathological findings were determined according to the classification of malignant tumors by the World Health Organization and International Union against Cancer Tumor-Node-Metastasis (TNM) staging system [18,19]. NSCLC tumor tissues and their matched non-tumor tissues were immediately frozen in liquid nitrogen and stored at −70°C until use. This study was approved by the Research Ethics Committee of Shanghai Chest Hospital. Informed consent was obtained from all of the patients. The clinicopathological features of patients are summarized in Table 1.

**Table 1:** **Correlation between lncRNA ANRIL expression and clinicopathological features in NSCLC patients**

**Parameters**	**Group**	**Total**	**lncRNA ANRIL**		***P value***
			**Low**	**High**	
Gender	Male	51	23	28	0.952
	Female	36	16	20	
Age (years)	<60	42	18	24	0.721
	≥60	45	21	24	
Tumor size (cm)	<3 cm	39	16	23	0.520
	≥3 cm	48	23	25	
Histology	Adenocarcinoma	38	20	18	0.197
	Squamous cell carcinoma	49	19	30	
TNM stage	I	26	19	7	0.001
	II-III	61	20	41	
Lymph node metastasis	Absence	40	32	8	0.000
	Presence	47	7	40	

### Cell culture and transfection

Three lung cancer cell lines (A549, SPC-A1, NCI-H1650) and a normal human bronchial epithelial cell line (16HBE) were purchased from the Institute of Biochemistry and Cell Biology of the Chinese Academy of Sciences (Shanghai, China). Cells were cultured in RPMI 1640 (Gibco) medium supplemented with 10% fetal bovine serum (FBS), 100 U/ml penicillin, and 100 mg/ml streptomycin in humidified air at 37°C with 5% CO_2_.

Lung cancer cells were transfected with either 50 nM siRNAs targeting ANRIL (si-ANRIL) or scrambled negative controls (si-NC) (GenePharma) using the Lipofectamine 2000 transfection reagent (Invitrogen) according to the instructions provided by the manufacturer. The target sequence for ANRIL siRNAs was 5′-GGUCAUCUCAUUGCUCUAU-3′ [13]. After 24 h, knockdown of ANRIL was confirmed via qRT-PCR.

### Cell proliferation assay

The in vitro cell proliferation of lung cancer cells was measured using the MTT method. In brief, cells were seeded into 96-well plates and transfected with 50 nM si-ANRIL or si-NC for 24 h. In the indicated time periods, 0.1 ml of spent medium was replaced with an equal volume of fresh medium containing MTT 0.5 mg/ml. Plates were incubated at 37°C for 4 hours, and then the medium was replaced with 0.1 ml of DMSO (Sigma) and plates were agitated at room temperature for 10 min. The absorbance was measured at 490 nm using an enzyme-labeled analyzer.

### Cell migration and invasion assays

Lung cancer cells were transfected with 50 nM si-ANRIL or si-NC. After 24 h, transfected cells were harvested. In migration assay, transfected cells (1x10^5^) were plated in the top chamber of Transwell assay inserts (Millipore) with a membrane containing pores with 8 mm diameters in 200 ml of serum-free RPMI1640. Assays were conducted in triplicate. Inserts were then placed into the bottom chamber wells of a 24-well plate containing RPMI1640 with 10% FBS as a chemoattractant. After 24 h of incubation, remaining cells were removed from the top layer of the insert by scrubbing with a sterile cotton swab. Invading cells from the bottom surface were stained with 0.1% crystal violet prior to being examined, counted and photographed using digital microscopy. Cell numbers were calculated in five random fields for each chamber, and the average value was calculated. In invasion assay, transfected cells (4x10^5^) were plated in the top chamber with a Matrigel-coated membrane. Bottom chambers were filled with conditioned medium. After a 48 h incubation period, the number of migrated cells on the lower side of the membrane was counted as described previously.

### Quantitative realtime PCR (qRT-PCR)

Total RNA was extracted from tissues or cultured cells using TRIzol reagent (Invitrogen). For qRT-PCR, RNA was reverse transcribed to cDNA by using a Reverse Transcription Kit (Takara). Real-time PCR analyses were performed with Power SYBR Green (Takara). Results were normalized to the expression of GAPDH. The PCR primers for ANRIL or GAPDH were as follows: ANRIL sense, 5′- TGCTCTATCCGCCAATCAGG-3′ and reverse, 5′-GGGCCTCAGTGGCACATACC-3′; GAPDH sense, 5′-GTCAACGGATTTGGTCTGTATT-3′ and reverse, 5′-AGTCTTCTGGGTGGCAGTGAT-3′. qRT-PCR and data collection were performed on ABI 7900. The relative expression of ANRIL was calculated and normalized using the 2^-ΔΔCt^ method relative to GAPDH.

### Statistical analysis

All statistical analyses were performed using SPSS version 18.0 software (IBM). Comparison of continuous data was analyzed using an independent *t*-test between the two groups, whereas categorical data was analyzed by the chi-square test. Overall survival curves were plotted according to the Kaplan-Meier method, with the log-rank test applied for comparison. A Cox proportional hazards regression analysis was used for univariate and multivariate analyses of prognostic values. The data are shown as the mean ± SD from at least three independent experiments. Values of P less than 0.05 were considered statistically significant.

## Results

### LncRNA ANRIL was up-regulated in NSCLC tissues

**Figure 1:**
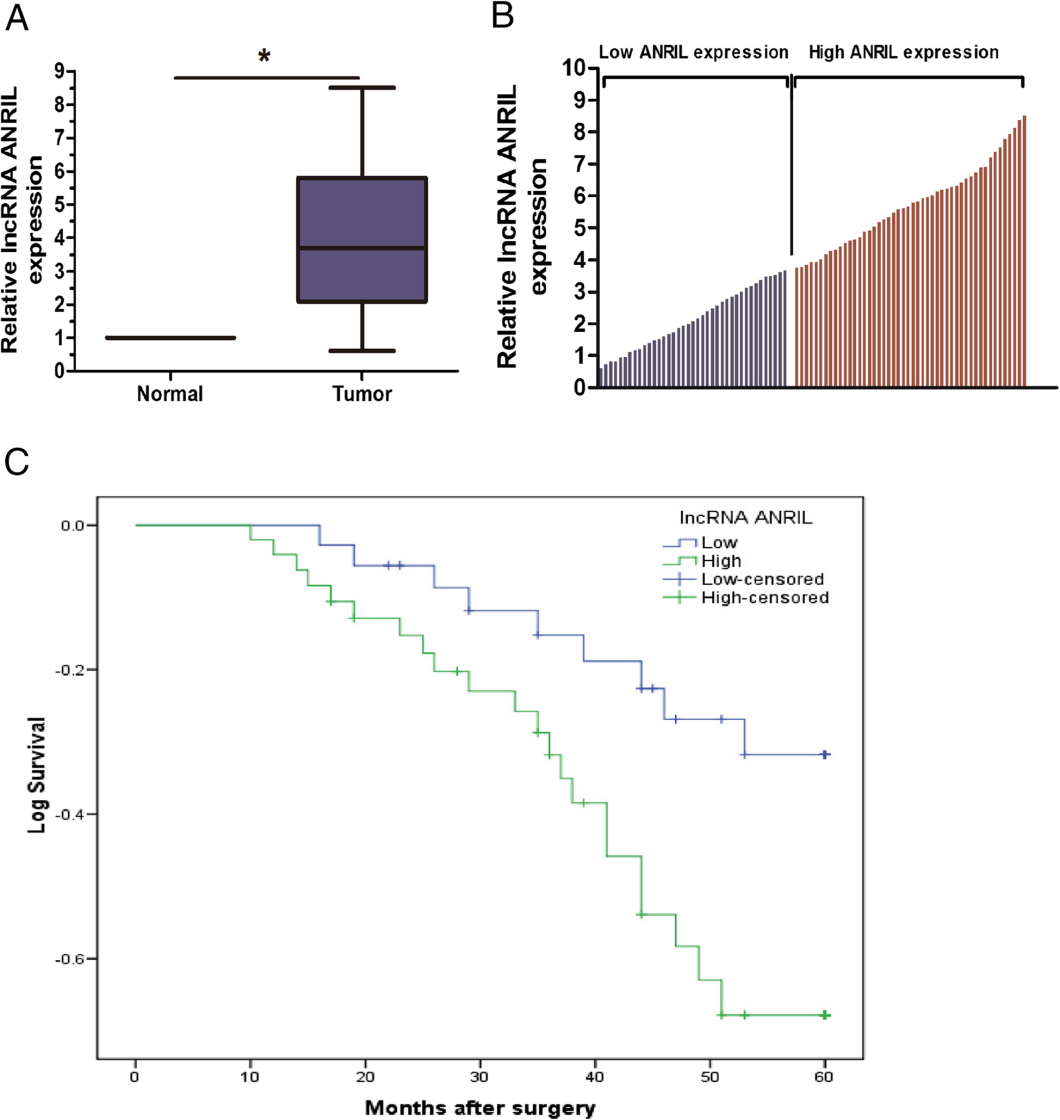
**Relative lncRNA ANRIL expression and its clinical significance in NSCLC patients. A**. Relative expression of lncRNA ANRIL in NSCLC tissues in comparison with adjacent non-tumor tissues. lncRNA ANRIL expression was examined by qRT-PCR and normalized to GAPDH expression. **B**. The 87 total NSCLC patients included in the study were divided into an relative high-ANRIL group (n = 48) and relative low-ANRIL group (n = 39) according to the mean value of relative lncRNA ANRIL expression. **C**. Kaplan-Meier overall survival curves according to lncRNA ANRIL expression level. Results are expressed as mean ± SD for three replicate determination. * P <0.05.

qRT-PCR assay was performed to detect the expression of lncRNA ANRIL in 87 NSCLC tissues and corresponding non-tumor tissues. As shown in Figure 1A, the relative level of lncRNA ANRIL expression was significantly higher in NSCLC tissues than in adjacent non-tumor tissues (P < 0.05). To assess the correlation of lncRNA ANRIL expression with clinicopathologic features, according to the mean value of relative lncRNA ANRIL expression (3.6) in tumor tissues, the 87 NSCLC patients were classified into two groups: relative high-ANRIL group: ANRIL expression ratio ≥ mean; relative low-ANRIL group: ANRIL expression ratio < mean (Figure 1B). As shown in Table 1, the expression of lncRNA ANRIL was significantly correlated with TNM stage and lymph node metastasis (P < 0.05). However, there were no significant correlations between lncRNA ANRIL expression and other clinicopathologic features including patient’s gender, age, tumor size and histology (P > 0.05). Taken together, these observations suggested that increased lncRNA ANRIL expression was associated with the progression and development of NSCLC.

### Over-expression of lncRNA ANRIL was associated with poor prognosis of NSCLC

Kaplan-Meier analysis and log-rank test were used to evaluate the effects of lncRNA ANRIL expression and the clinicopathological features on overall survival of NSCLC patients. As shown in Figure 1C, the overall survival of patients with high lncRNA ANRIL expression was significantly poor than that of those with low lncRNA ANRIL expression (P < 0.05). Univariate analysis showed that TNM stage, lymph node metastasis and lncRNA ANRIL expression were significantly correlated with poor overall survival of NSCLC patients (P < 0.05; Table 2). Multivariate analysis suggested that relative lncRNA ANRIL expression level, TNM stage and lymph node metastasis were independent prognostic indicators for the overall survival of NSCLC patients (Table 2). These results revealed that lncRNA ANRIL expression could develop as a powerful independent factor for predicting the prognosis of NSCLC patients.

**Table 2:** **Univariate and multivariate analysis of prognostic parameters in NSCLC patients by Cox regression analysis**

**Variable**	**Univariate analysis**			**Multivariate analysis**		
	**Risk ratio**	**95% CI**	***P-value***	**Risk ratio**	**95% CI**	***P-value***
Gender	1.328	0.653-2.344	0.552			
Male vs female						
Age (years)	1.817	0.726-3.408	0.472			
≥60 vs <60						
Tumor size	1.533	0.874-1.912	0.764			
≥3 cm vs <3 cm						
Histologic grade	0.892	0.531-1.275	0.318			
Squamous cell carcinoma vs adenocarcinoma						
TNM stage	2.936	1.526-5.712	0.007	2.517	1.368-5.215	0.011
II-III vs I						
Lymph node metastasis	3.371	1.484-6.933	<0.001	2.868	1.392-6.173	0.006
Presence vs absence						
lncRNA ANRIL	2.793	1.425-5.791	0.002	2.538	1.374-5.452	<0.001
High vs low						

### lncRNA ANRIL expression in lung cancer cells and lung cancer cell transfection

**Figure 2:**
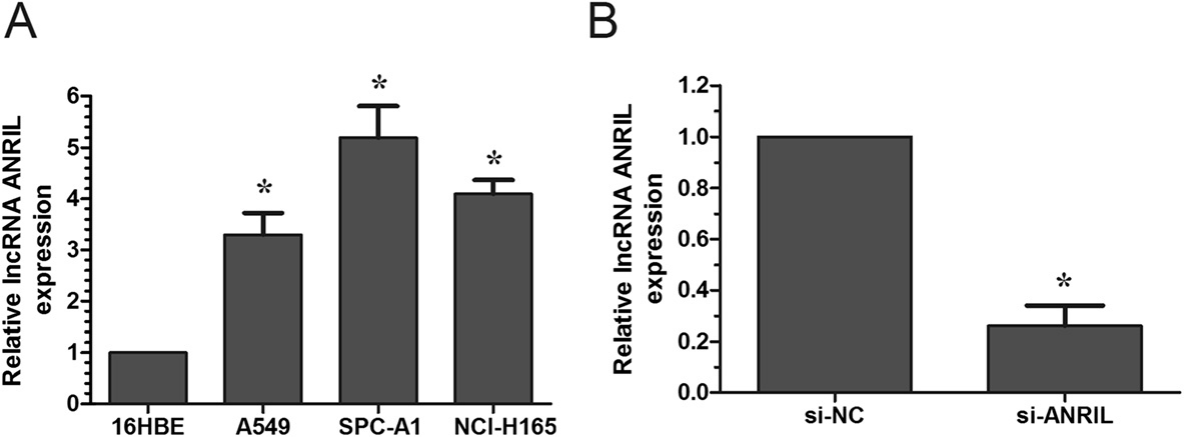
**Relative lncRNA ANRIL expression level in lung cancer cells. A**. Analysis of lncRNA ANRIL expression levels in NSCLC cell lines (A549, SPC-A1, NCI-H1650) compared with the normal bronchial epithelial cell line (16HBE) by qRT-PCR. **B**. Analysis of lncRNA ANRIL expression following treatment of SPC-A1 cells with si-ANRIL or si-NC by qRT-PCR. Results are expressed as mean ± SD for three replicate determination. * P <0.05.

To investigate the roles of lncRNA ANRIL in NSCLC, we performed qRT-PCR to evaluate the levels of ANRIL in three NSCLC cell lines and one normal human bronchial epithelial cell line (16HBE). The expression of ANRIL was higher in all three cancer cell lines compared with the levels observed in 16HBE cells, with the highest in SPC-A1 cells (Figure 2A). Thus SPC-A1 cells were selected and transfected with si-ANRIL or si-NC. Our results showed that lncRNA ANRIL expression was effectively knocked down in SPC-A1 cells (Figure 2B).

### Knockdown lncRNA ANRIL suppressed lung cancer cell growth and metastasis

**Figure 3:**
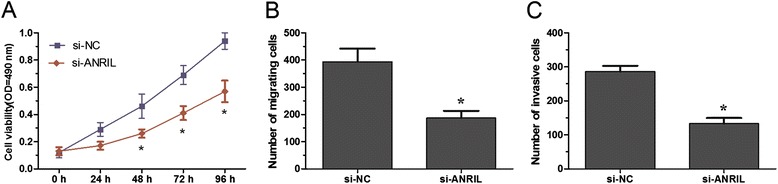
**Down-regulation of lncRNA ANRIL promotes lung cancer cell proliferation, migration and invasion. A**. MTT assay was performed to determine the proliferation of si-ANRIL transfected SPC-A1 cells. **B**. Transwell migration assay was used to investigate the migration of si-ANRIL transfected SPC-A1 cells. **C**. Transwell invasion assay was performed to investigate the invasion of si-ANRIL transfected SPC-A1 cells. Results are expressed as mean ± SD for three replicate determination. * P <0.05.

We investigated the impact of lncRNA ANRIL on the growth of lung cancer cells, MTT assay showed that the proliferation rate of of SPC-A1 cells transfected with si-ANRIL was significantly decreased compared with the si-NC group (P < 0.05, Figure 3A). Next, Transwell migration and invasion assays were used to examine the effect of lncRNA ANRIL on lung cancer cell metastasis. Transwell migration assay showed that the migration ability of SPC-A1 cells transfected with si-ANRIL was significantly decreased compared with the si-NC group (P < 0.05, Figure 3B). Transwell invasion assay revealed that the invasion capacity of SPC-A1 cells transfected with si-ANRIL was notably down-regulated compared to si-NC group (P < 0.05, Figure 3C). These results suggested that lncRNA ANRIL could promote the growth and metastasis of lung cancer cells.

## Discussion

The sequencing of the human genome revealed that the coding portion of the genome represents less than 2% of the genome. The remaining 98% of transcription products of the genome consists of non-coding RNA sequences, including microRNAs and lncRNAs [20]. Recently, numerous pieces of evidence showed that dysregulation in lncRNAs are proved to contribute in tumor development in many cancer types and can be used to develop as biomarkers and therapy target [21]. For example, Zhang et al. demonstrated that lncRNA SPRY4-IT1 (SPRY4 intronic transcript 1) was increased in clear cell renal cell carcinoma (ccRCC) tissues and ccRCC patients with higher SPRY4-IT1 expression had an advanced clinical stage and poorer prognosis [22]. Wang et al. showed that lncRNA PlncRNA-1 was significantly higher in human esophageal squamous cell carcinoma and correlated with advanced clinical stage and lymph node metastasis. Knockdown of PlncRNA-1 reduced cell proliferation and increased the apoptosis in vitro [23]. Sun et al. found that GAS5 (Growth arrest-specific 5) expression was markedly down-regulated in gastric cancer and associated poorer disease-free survival and overall survival of gastric cancer patients. Moreover, ectopic expression of GAS5 was demonstrated to decrease gastric cancer cell proliferation and induce apoptosis in vitro and in vivo [24]. However, the expression profile and potential function of lncRNA ANRIL in NSCLC is still unknown.

In the study, we explored the clinical significance of lncRNA ANRIL in NSCLC patients for the first time. By using qRT-PCR, we found that lncRNA ANRIL was increased in NSCLC tissues and lung cancer cell lines to a greater extent than in adjacent non-tumor tissues and normal human bronchial epithelial cell line. We also revealed that the relative expression level of lncRNA ANRIL was associated with TNM stage and lymph node metastasis of NSCLC patients. These findings suggested that a higher level of lncRNA ANRIL expression may be involved in NSCLC pathogenesis and progression.

Furthermore, we analyzed a correlation between lncRNA ANRIL expression level and prognosis of NSCLC. Our results showed patients with high lncRNA ANRIL expression had a shorter overall survival rate than those with low lncRNA ANRIL group. These findings were further supported by the univariate and multivariate analyses of Cox proportional hazards regression model, indicating that the expression of lncRNA ANRIL could be an independent factor for predicting the prognosis of NSCLC patients. Therefore, our data demonstrated that increased expression of lncRNA ANRIL was associated with an high risk of death from NSCLC. Then, we analyzed the effect of lncRNA ANRIL expression on the growth and metastasis of lung cancer cells. We found that down-regulated expression of lncRNA ANRIL significantly decreased proliferation, migration and invasion capability of lung cancer cells in vitro. These data further support the importance of ANRIL in cellular biology and oncogenesis of lung cancer cells and indicate that ANRIL is involved in the development and progression of NSCLC.

## Conclusion

In summary, our study demonstrated that the expression level of lncRNA ANRIL was increased in NSCLC tissues compared to that in the adjacent non-tumor tissues. Elevated lncRNA ANRIL expression has been associated with poor prognosis of overall survival, likely due to the ability of lncRNA ANRIL to promote cell growth and metastasis in lung cancer cells. These results indicated that lncRNA ANRIL played a critical role in the progression of NSCLC. The development of ANRIL-based therapeutic strategies for the downregulation of such oncogenic lncRNAs may provide a novel and promising alternative therapeutic approach for future cancer treatment.

## Abbreviations

lncRNAs: Long non-coding RNAs
ncRNA: Non-coding RNA
NSCLC: Non-small cell lung cancer
ccRCC: Clear cell renal cell carcinoma
TNM: Tumor-node-metastasis
DMSO: Dimethyl sulfoxide
FBS: Fetal bovine serum
ANRIL: Antisense non-coding RNA in the INK4 locus
SPRY4-IT1: SPRY4 intronic transcript 1
GAS5: Growth arrest-specific 5
siRNA: Small interfering RNA
si-ANRIL: siRNAs targeting ANRIL
si-NC: Scrambled negative control
qRT-PCR: Quantitative real-time PCR
GAPDH: Glyceraldehyde-3-phosphate dehydrogenase
MTT: 3-(4,5)-dimethylthiahiazo (−z-y1)-3,5-di- phenytetrazoliumromide
PRC2: Polycomb repressive complex 2
